# Clinico-pathological and transcriptomic determinants of SLFN11 expression in invasive breast carcinoma

**DOI:** 10.1186/2051-1426-3-S1-O3

**Published:** 2015-08-14

**Authors:** Gabriele Zoppoli, Sylvain Brohee, Christine Desmedt, Christos Sotiriou, Alberto Ballestrero

**Affiliations:** 1 Department of Internal Medicine (DiMI), University of Genova, Genova, Italy; 2 Breast Cancer Translational Research Laboratory J.C. Heuson, Institut Jules Bordet, Bruxelles, Belgium

## 

SLFN11 is a putative DNA/RNA helicase we discovered as causally associated with sensitivity to DNA damaging agents, such as platinum salts, topoisomerase I and II inhibitors, and other alkylators in the NCI-60 panel of cancer cell lines [1]. Later, SLFN11 was identified as an early interferon response gene, in association with HIV infection [2]. Here we assessed SLFN11 determinants in a gene expression meta-set of 5,061 breast cancer patients annotated with clinical data and multigene signatures obtained with the package genefu [3]. By correlation analysis, we found 537 transcripts above the 95th percentile of Pearson’s coefficients with SLFN11, identifying “immune response”, “lymphocyte activation”, and “T cell activation” as top Gene Ontology enriched processes [4]. Through multiple correspondence analysis, we discovered a subgroup of patients characterized by high SLFN11 levels, ER negativity, basal phenotype, elevated CD3D, STAT1 signature [5], and young age. Fitting a penalized maximum likelihood lasso regression model [6], we found a strong multivariable association of SLN11 with the stroma 1 and stroma 2 signatures [7,8], associated with basal cancer and response to chemotherapy in ER- tumors. Finally, using Cox proportional hazard regression, ER-, high proliferation, high SLFN11 patients undergoing chemotherapy treatment showed a significantly longer disease-free interval than other patient categories included in our model.

## References

[B1] Zoppoli G Regairaz M Leo E Reinhold WC Varma S Ballestrero A Doroshow JH Pommier Y Putative DNA/RNA helicase Schlafen-11 (SLFN11) sensitizes cancer cells to DNA-damaging agents Proc Natl Acad Sci U S A 2012 109 37 15030 5 10.1073/pnas.1205943109 22927417 PMC3443151

[B2] Li M Kao E Gao X Sandig H Limmer K Pavon-Eternod M Jones TE Landry S Pan T Weitzman MD David M Codon-usage-based inhibition of HIV protein synthesis by human schlafen 11 Nature 2012 491 7422 125 8 10.1038/nature11433 23000900 PMC3705913

[B3] Haibe-Kains B Schroeder M Bontempi G Sotiriou C Quackenbush J genefu: Relevant Functions for Gene Expression Analysis, Especially in Breast Cancer 2014 R package version 1.16.0

[B4] Huang da W Sherman BT Lempicki RA Systematic and integrative analysis of large gene lists using DAVID bioinformatics resources Nat Protoc 2009 4 1 44 57 19131956 10.1038/nprot.2008.211

[B5] Desmedt C Haibe-Kains B Wirapati P Buyse M Larsimont D Bontempi G Delorenzi M Piccart M Sotiriou C Biological processes associated with breast cancer clinical outcome depend on the molecular subtypes Clin Cancer Res 2008 14 16 5158 65 10.1158/1078-0432.CCR-07-4756 18698033

[B6] Friedman J Hastie T Tibshirani R Regularization Paths for Generalized Linear Models via Coordinate Descent J Stat Softw 2010 33 1 1 22 20808728 PMC2929880

[B7] Farmer P Bonnefoi H Anderle P Cameron D Wirapati P Becette V AndrÃ© S Piccart M Campone M Brain E Macgrogan G Petit T Jassem J Bibeau F Blot E Bogaerts J Aguet M Bergh J Iggo R Delorenzi M A stroma-related gene signature predicts resistance to neoadjuvant chemotherapy in breast cancer Nat Med 2009 15 1 68 74 10.1038/nm.1908 19122658

[B8] Finak G Bertos N Pepin F Sadekova S Souleimanova M Zhao H Chen H Omeroglu G Meterissian S Omeroglu A Hallett M Park M Stromal gene expression predicts clinical outcome in breast cancer Nat Med 2008 14 5 518 27 10.1038/nm1764 18438415

